# Impact of PRECEDE–PROCEED Model Audits in Cancer Screening Programs in Lombardy Region: Supporting Equity and Quality Improvement

**DOI:** 10.3390/curroncol31100445

**Published:** 2024-10-03

**Authors:** Stefano Odelli, Margherita Zeduri, Maria Rosa Schivardi, Davide Archi, Liliana Coppola, Roberto Genco Russo, Maristella Moscheni, Elena Tettamanzi, Fabio Terragni, Michela Viscardi, Valentina Vitale, Anna Odone, Danilo Cereda, Silvia Deandrea

**Affiliations:** 1 General Directorate of Welfare of the Lombardy Region, 20124 Milan, Italy; odelli.stefano@hsr.it (S.O.); margherita.zeduri01@universitadipavia.it (M.Z.);; 2 School of Public Health, University Vita-Salute San Raffaele, 20132 Milan, Italy; 3 Department of Public Health, Experimental and Forensic Medicine, University of Pavia, 27100 Pavia, Italy; 4 Control Agency of the Lombardy Healthcare System (ACSS), 20124 Milan, Italy; 5 Prevention Department, Health Protection Agency of Pavia, 27100 Pavia, Italy

**Keywords:** PRECEDE–PROCEED model, cancer screening, audit, Lombardy region, equity, quality improvement

## Abstract

Background: Health disparities related to socio-economic factors impact access to preventive health interventions. The PRECEDE–PROCEED model, a multidimensional approach to health promotion, has been adapted to optimise cancer screening programs in Lombardy, Italy, addressing these disparities. Methods: This study evaluated the application of systemic audits based on the PRECEDE–PROCEED model across Lombardy cancer screening programs. A systematic region-wide audit was performed in 2019, and follow-up audits were performed in 2022–2023. Data were collected using structured analysis methodologies, including epidemiological, behavioural, and organisational assessments. Results: The 2019 audit showed strengths in participation and quality standards but identified challenges in cervical cancer screening coverage and waiting times for assessments. Improvements plans included the digitisation of processes and stakeholder engagement. The 2022–2023 audits reported increased coverage for breast and colorectal screenings, but a slight decline in participation rates and examination coverage. Organisational improvements were noted, yet gaps in training and equity-targeted actions remained. Conclusion: The PRECEDE–PROCEED model audits led to notable improvements in the quality and equity of cancer screening programs in Lombardy. Sustained focus on digital integration, continuous re-training, and targeted equity interventions is essential for further progress.

## 1. Introduction

International organisations such as the World Health Organisation and the European Union place a growing emphasis on the social determinants of health [1,2]. In fact, individuals facing social disadvantages often exhibit higher mortality rates, increased incidence of illness, prolonged hospitalisations, and diminished mental and physical well-being. These disparities tend to decrease in a linear fashion as individuals transition into higher socio-economic classes [3,4,5,6,7,8]. Socio-economic disparities significantly impact access to preventive interventions and effective medical–surgical treatments. Preventive health interventions, such as cancer screening programs, demonstrate efficacy, but addressing health inequalities requires a proactive, initiative-driven approach [9,10].

In accordance with the Italian legal framework, the implementation of breast, colorectal and cervical cancer screening programs is mandatory across all regional healthcare systems within Italy; the government includes them in the Essential Levels of Assistance (LEAs), establishing a mandatory basic service provision that every region should achieve. However, the implementation of these programs can vary across different regions due to differences in healthcare infrastructure, resource allocation, and population demographics. Moreover, after meeting these obligatory standards, regions have the autonomy to expand services in accordance with scientific evidence and resource availability.

The PRECEDE–PROCEED model provides a comprehensive framework for evaluating health promotion initiatives and is well suited to cancer screening programs [11]. The PRECEDE phase identifies predisposing, reinforcing, and enabling factors that affect screening participation, such as knowledge, attitudes, healthcare access, and policy support, helping in understanding barriers. The PROCEED phase involves implementing interventions and assessing their effectiveness in improving screening uptake, early detection rates, and reducing cancer morbidity and mortality [11]. The PRECEDE–PROCEED methodology has been considered an effective tool to shape the implementation of screening programs within the framework of the National Prevention Plan 2010–2013 because of its multidimensional and multidisciplinary approach to health promotion. The model is widely used to support the process of planning and monitoring actions, assuming that health and health behaviours are influenced by multiple factors—epidemiological, socio-psychological, administrative, political, and environmental—which must be considered and assessed for their modifiability in order to ensure effective interventions. Through a project supported by the Ministry of Health aimed to develop strategies to improve participation in screening, the PRECEDE–PROCEED model was adapted as an operational analysis grid for use in screening programs [12]; in 2015, the Lombardy region was one of the six regions engaging in a pilot project exploring the health equity audit methodology [11]. Building upon the pilot project’s outcomes, Lombardy moved to systematise the methodology and address identified challenges through expert-led revisions. These efforts led to the activation of participatory audit pathways, aiming to bolster adherence and engage socio-culturally vulnerable populations.

Following this initial experience, in 2019, the Lombardy region systematically applied the methodology to conduct audits across all its local health authorities, renamed into Health Protection Agencies (HPAs) after the 2016 reform [13]. This systematic approach aimed to further refine and optimise screening programs, enhancing healthcare access and quality for all citizens. A second audit cycle, intended to monitor the implementation of improvement actions, was scheduled for 2020. However, due to the COVID-19 pandemic, it was postponed and carried out between 2022 and 2023.

The aim of this study is to provide evidence of improved results achieved over the years through the application of systemic audits for cancer screening programs.

## 2. Materials and Methods

### 2.1. Cancer Screening Programs in Lombardy Region

Lombardy is the most densely populated region in Northern Italy, with approximately 10 million residents. Its regional healthcare system is characterised by a clear demarcation between the planning, coordinating, and financing of services managed by the HPAs, and healthcare providers such as hospitals and outpatient clinics. The Directorate General for Welfare of the Lombardy Region (DGW-LR) guarantees the implementation of population-based cancer screening programs through the HPAs. The Lombardy region has extended its screening services beyond the LEA requirements, offering mammographic screening to women aged 45 to 74 and faecal occult blood testing for colorectal cancer prevention in both men and women aged 50 to 74. Additionally, the Lombardy region has adopted a differentiated protocol for HPV testing based on vaccination status: unvaccinated women are offered triennial pap tests between the ages of 25 and 29, followed by HPV testing for women aged 30 to 64, regardless of vaccination status. Following a national directive, since 2022, the Lombardy region has also been offering Hepatitis C Virus (HCV) screening for all individuals born between 1969 and 1989, initially only for those attending hospitals and outpatient blood collection centres [14]. Those with positive anti-HCV are offered HCV–RNA testing and positive cases, eradication therapy [15].

### 2.2. Lombardy Audit Model: 2019 Cycle

During 2019, the screening audit focused on the HPAs and the audit team included a regional official and an expert auditor chosen among specifically trained healthcare professionals from one of the other eight HPAs. The analysis of the screening program using the PRECEDE–PROCEED model was performed using a custom Microsoft Access macro programmed to automate the compilation of reports [12]. The audit included epidemiological (quality indicators from the three screening programs) [16], behavioural, and organisational analysis, and then the study of nine thematic areas: software, programming, invitation, delivery, training, quality, collaborations, communication, and equity. Each thematic area corresponds to actions by which the eight HPAs were assessed in terms of frequency of application (‘always’, ‘most of the time’, ‘occasionally’, or ‘never’). For each action among those not implemented or those implemented with critical issues, the auditors and the audited parties identified sustainability, priorities, and elaborated solutions for each critical issue. The audit ended with a draft of the final report and an improvement plan, subsequently subjected to periodic monitoring.

### 2.3. Audit Cycle 2022/2023

A second audit cycle, aimed at monitoring the implementation of improvement plans, was scheduled for 2020. However, due to the COVID-19 pandemic, the improvement actions were suspended until 2022. A new cycle of audits was eventually performed in late 2022 and 2023. The new audit cycle included two risk management methodologists from the Lombardy Healthcare System Control Agency (ACSS) in the audit team and it also assessed the regional screening program for the elimination of the HCV [15]. For the first time, HPAs were jointly audited with a sample of their healthcare facilities in order to promote a participatory approach among the actors included in the screening process and to facilitate the establishment of joint actions. The audit followed a structured analysis methodology, with a preliminary assessment of indicators and procedures. The audit included interviews and on-site visits with a random sampling of activities and a review of documentation. During the analysis of the audited facility’s organisational structure, a list of activities and processes —varying in number between HPAs— was compiled, and a random selection of these was chosen for auditing. A checklist, developed from the items assessed in the 2019 cycle, was used as a guiding tool for conducting the audit: it contained information on the previous nine thematic areas and included a new section devoted to the new HCV screening. Some questions were entirely open-ended, while others were open-ended but pre-coded. Other questions used a scaled format. Many of the questions were qualitative and were subsequently evaluated using a 0 to 3 scoring scale (with 3 being the best possible score) based on their level of alignment with regional and national goals and guidelines.

### 2.4. Data Analysis

In both audit cycles, the answers of the individual HPAs and evidence collected for the nine thematic areas were converted into a quantitative form to determine the percentage of achievement per area, with 100% indicating that all actions were fully implemented. When the questions from the 2019 cycle had already been met, they were replaced by more challenging ones, making direct comparisons between the 2019 and 2022/2023 scores not possible. Moreover, since a different audit methodology was applied in 2022/2023, the results of the epidemiological analysis focused on each HPA were not conducted for these years. Therefore, in order to assess the possible impact of the audit cycle, we conducted a comparison between the epidemiological indicators collected at the regional level between 2019 and 2022.

This study used aggregated epidemiological data and system performance responses, without collecting individual-level or identifiable data. The project was exempt from Institutional Review Board (IRB) review, as it focused on quality improvement and institutional practices.

## 3. Results

### 3.1. Outcomes of the Epidemiological and Behavioural Analysis—2019 Cycle

In Table 1, the indicators collected for the HPA epidemiological analysis were reported together with their respective reference standards [17,18,19,20,21]. For colorectal and mammographic screenings, the age cohorts 50–74 were analysed. Cervical screening data refer to women aged 25 to 64.

The major challenge identified was the lack of activation of the cervical cancer screening program. Specifically, only four HPAs reported cervical cancer screening data, and, in some cases, the program was implemented only in selected areas. Another critical issue across most HPAs was waiting times, especially for colorectal cancer, where the goal of >90% of colonoscopies performed within 30 days of a positive faecal occult blood test result was not achieved by all eight HPAs. The complete colonoscopy rate was excellent for all HPAs.

Regarding breast screening in particular, the percentage of T2+ stages at the subsequent exams was higher than the <25% target for five out of eight HPAs, while the rate of further investigations for first examinations fell short of the <7% target for seven out of eight HPAs. On the other hand, strengths lay in screening coverage and participation indicators.

### 3.2. Outcomes of Organisational Analysis—2019 Cycle

**Figure 1:**
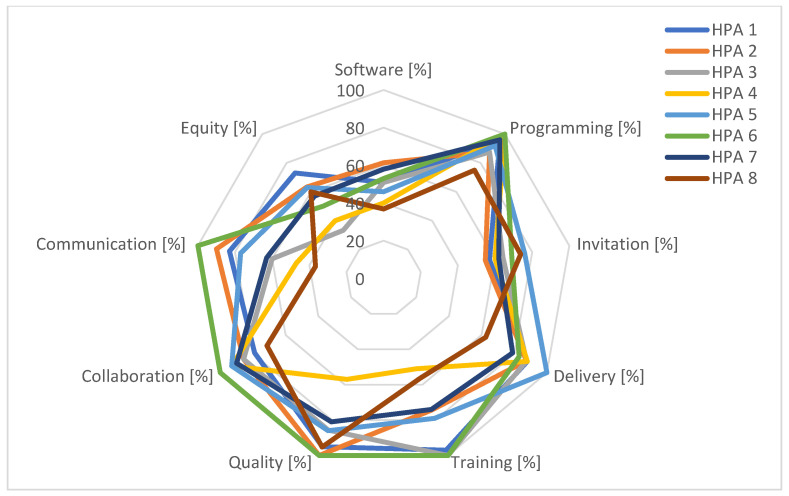
Percentages of achievement of target by thematic area for each HPA, 2019 audit cycle.

**Figure 2:**
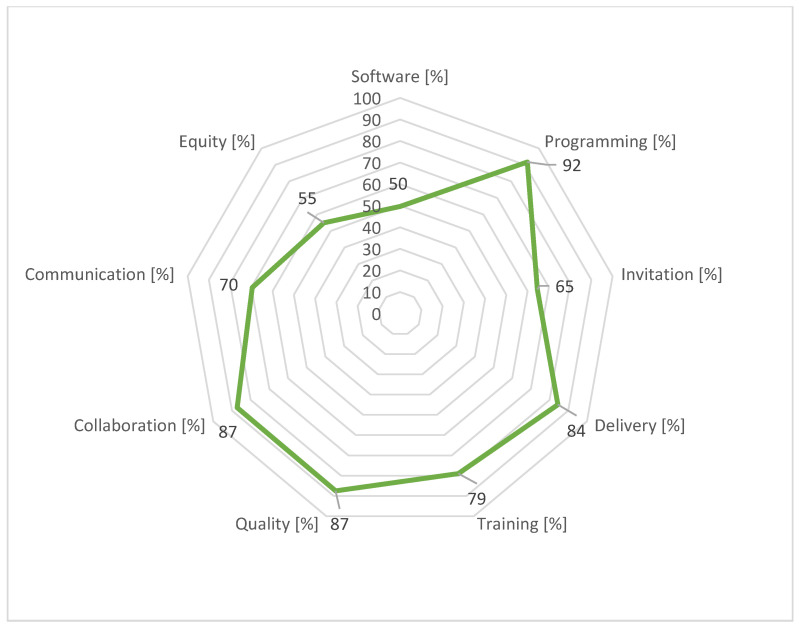
Average of percentages of achievement of target by thematic area for Lombardy region, 2019 audit cycle.

Figure 1 and Figure 2 contain radar charts highlighting the strengths and weaknesses of the Lombardy screening programs.

The software used by the eight HPAs facilitated scheduling calls for screening tests; however, not all software allows General Practitioners’ (GPs’) access to the cancer screening registry. Suboptimal scores were related to the import of results for screening and assessment tests, for breast, colorectal, and cervical programs, due to the lack of an IT integration between screening centres and providers. Unlike laboratories for faecal immunochemical tests, in many HPAs, the radiological and pathology reports were not fully digitised and, in some instances, were still used in paper format.

Regarding programming, all eight HPAs met the expectations concerning organisational procedures, their application, and the timely annual reporting of activities (scores >75% for all HPAs and a regional average score of 92%). Challenges were stakeholder involvement and, in some cases, the lack of procedures for monitoring activities.

There were discrepancies in invitation activity among HPAs, mostly attributable to different internal organisational methods and software set-ups. The HPAs’ scores ranged from a minimum of 55% to a maximum of 76%, with an average of 65%. All invitations and reminders were performed by paper letter, while notification of a positive screening test was made by telephone. For all HPAs, there was a toll-free number dedicated to citizens. The pre-invitation exclusion criteria (e.g., recent screening tests outside the program) met the regional guidelines for all HPAs.

Performance in the delivery domain showed a uniform quality level of service throughout the regional territory.

All the HPAs had a training plan for screening personnel and two out of eight HPAs reached the maximum score. Participation in training events involved various professional roles such as radiographers, radiologists, pathologists, laboratory technicians, obstetricians, endoscopists, gynaecologists, and GPs.

Regarding quality, the annual reporting of screening activity data was promptly carried out by the majority of HPAs.

Screening centres and external entities (research institutions, universities, GPs, pharmacies, and public and private providers) were involved in defining the communication projects for all eight HPAs. Although projects included updating websites, social media pages easily accessible to users were rarely in place and two HPAs reported a score of <50%. All screening centres systematically collaborated with other HPA departments, including the administrative area, the risk management unit, the public relations office, primary care, the cancer registry, the health promotion unit, and information systems.

The analysis of equity revealed, for the majority of HPAs, a lack of activities. Equity represented the thematic area with the lowest average score together with the software. In this regard, the improvement plans of the majority of HPAs included the enhancement of outreach activities for vulnerable subjects.

### 3.3. Outcomes of the Epidemiological Analysis—2022 Cycle

Table 2 shows a comparison between the indicators in 2019 and 2022. The target population increased for all programs due to demographic factors, but primarily due to changes in the regional protocol, with an extension to 50–74 years for breast and colorectal cancer screening, compared to the 50–69 years national target. For cervical cancer screening, the implementation of the differentiated protocol for testing based on primary HPV started in 2019, but the HPAs were still in a gradual roll-out phase [20,21,22].

As the target population grew, so did the number of invitations. The adjusted invitation coverage in 2022 remained excellent for breast and colorectal cancer screening, above the standard of 95%. For cervical screening, as mentioned, the coverage increased over the years, in step with the involvement of all territories and the entire eligible regional population.

The adjusted participation rate decreased by a few percentage points for all screening programs. The coverage by examination slightly decreased for breast and colorectal cancer screening, while it increased significantly for cervical screening. The cancer detection rates remained about the same compared to 2019.

### 3.4. Outcomes of Organisational Analysis—2022 Cycle

**Figure 3:**
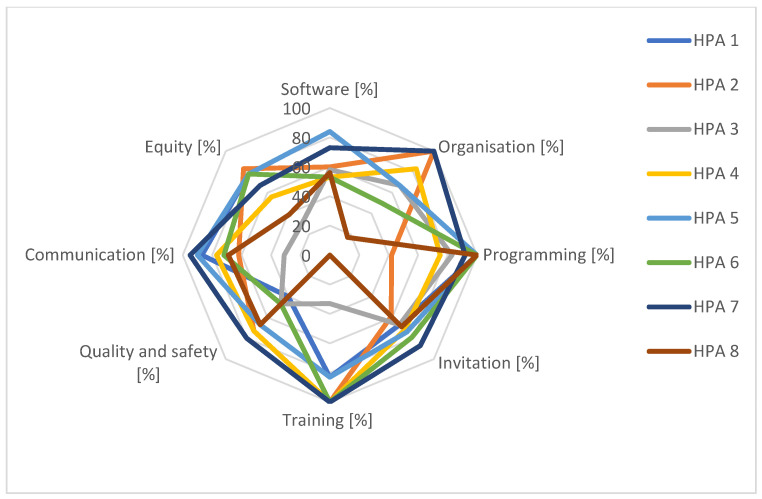
Percentages of achievement of target by thematic area for each HPA, 2022/2023 audit cycle.

**Figure 4:**
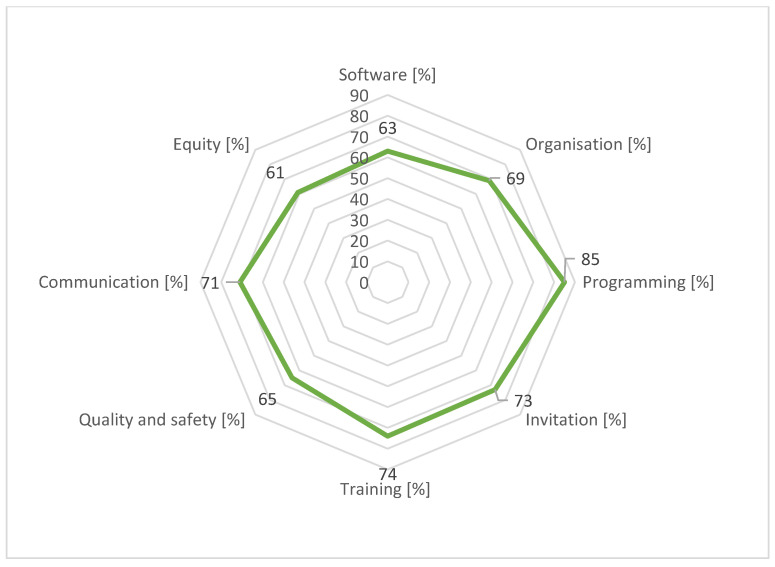
Average of percentages of achievement of target by thematic area for Lombardy region, 2022/2023 audit cycle.

Figure 3 and Figure 4 contain radar charts of the second audit cycle. In 2022/2023, the overall software performance improved and, for an additional HPA, integration with software used by the GPs was developed. However, the generation of invitations was not yet a straightforward process for all HPAs and the software thematic area had the lowest regional average score (61%).

With regard to organisation, again, most screening activities were based on written procedures and just one HPA reported an average score <50% for this area. However, for some HPAs the planned updates of these procedures had not been carried out. Furthermore, for some procedures, the inclusion of measurable indicators, reference standards, and responsibility for monitoring were still missing. During the audits, it was also recommended to write shared operational procedures between HPAs and providers. For those HPAs for which there was a critical issue in terms of human resource allocations, which had an important impact on screening activities, the organisation of the HPAs showed a strong inclination to build a flexible system to guarantee both the quality and the supply of screening.

By 2023, all HPAs reported evidence of an annually published report shared with stakeholders, with fruitful integrations between HPAs and healthcare facilities; there is room for improvement in the choice of indicators to use for monitoring. No significant changes were noted with regards to pre-invitation exclusion procedures.

Training programs could still be improved, especially by extending the offer to GPs, pharmacy staff, and other stakeholders. Two HPAs reported scores of <50% but the remaining performed better, with scores >80%; four HPAs achieved the maximum score.

In the area of quality and safety, the auditors recommended improving the risk management of screening pathways, favouring the reporting of sentinel events, adverse events, or near misses. In some HPAs, there was a lack of dedicated risk management and adverse event management procedures; in another one, despite their presence, not all staff members demonstrated awareness of them. The average score for this thematic area was around 60%. The ability to report adverse events within the corporate incident reporting system is essential for all HPAs and further efforts are needed to achieve better results.

With regard to communication, most of the websites were up to date, but for some HPAs and providers, it was suggested that communications regarding the HCV screening program should be strengthened. The dissemination of programs via social media needs to be strengthened. Only one HPA reported an insufficient score.

In 2023, thanks to the launch of the regional project for the profiling of the vulnerable population [23,24], two out of eight HPAs initiated specific equity-oriented activities and others outlined targeted objectives to be realised in the future. The target populations were prison inmates and nursing home residents.

With regard to the new HCV campaign, the correct use of regional communication/information material was noted [25], but also the absence of procedures governing the specific screening pathway for three HPAs. The low level of advancement of HPAs on this issue did not allow the quantitative evaluation of the answers to these questions.

## 4. Discussion

The outcomes of the participatory audit conducted in 2019 for the eight HPAs of the Lombardy region show that it allowed HPAs to identify concrete improvement actions. The participatory audit process highlighted local peculiarities and the importance of a screening program, consistent with overall goals but tailored to local specificities. Programming, quality, and collaboration thematic areas reported excellent results for both audit cycles with a qualitative uniformity of services throughout the regional territory. The two weakest thematic areas in the 2019 audit were software and equity, both of which scored poorly regionally.

The criticality leading to the heterogeneity of the screening software use between all the HPAs emerged significantly during the 2019 audit cycle. Thanks to the drafting of improvement plans and the implementation of effective programs, in the following years, the functionality of the software improved. DGW-RL is already planning the implementation of a new single regional management system that would improve integration between the agendas of screening centres and providers. It would also be an important strength, as it could resolve much of the diversity in favour of more efficient communication integration and more seamless and secure governance. As a result, regional scores increased by 13%, reaching sufficiency. In addition, several innovative apps have been tested in recent years, such as the colorectal cancer campaign with specific information and health education that could be used by all citizens [26].

Also, the equity area revealed a lack of projects, just partially reported during the following audit. For this reason, in 2023, a new regional analysis project for the profiling of the fragile population was conducted and all HPAs were mandated to implement a targeted equity plan containing objectives and strategic lines to be applied in order to reduce health inequalities. In agreement with an equity lens approach, in Italy, various national health planning documents such as the National Prevention Plan 2020–2025 address health inequalities as a fundamental principle and intervention priority, to be translated into specific actions within central actions and regional prevention plans [27]. In order to reduce the health gap between individuals from different socio-economic classes, in addition to addressing the direct determinants of health inequalities (such as income distribution, education, and the labour market), interventions can be implemented targeting the intermediate determinants of health, including lifestyles, health behaviour, and access to prevention, diagnosis, and treatment services. Therefore, prevention and health promotion could become key tools for the health system to tackle health inequalities [28]. Many of the target risk factors of the National Prevention Plan exhibit exposure inequalities that explain a significant proportion of mortality inequalities, justifying an equity audit investment in their respective application projects within the Regional Prevention Plan. The latter transforms equity in prevention from an inspiring principle to an operational method for guiding stakeholders’ choices and actions. This is in line with the global priority to prevent and counteract health inequalities.

With regard to the outcomes of the epidemiological analysis, in the 2022/2023 cycle, there was an improvement in the number of invitations and tests carried out for the three screening lines. The decrease in a few percentage points in the adjusted participation rate for the three screening programs was due to the reduced participation of the population in the screening offer in the post-COVID-19 period compared to the pre-pandemic period, in line with what has been observed at the national level [29]. Furthermore, while older age is considered to be a factor favouring participation, the presence of multiple comorbidities is a risk factor for non-participation in breast and colorectal cancer screening programs, providing further explanations for the decline in adjusted participation rates as the target population expands [25]. In 2019, only half of the Health Protection Agencies (HPA) of Lombardy had activated a cervical screening program, and although an improvement was reported in 2022–2023, the figures were much lower than those for breast and colorectal programs. This is partly due to the strong presence of private healthcare facilities in Lombardy, which have historically created parallel channels for spontaneous prevention, not accounted for in regional data. Also the COVID-19 pandemic surely played a role [30]. To improve participation, in 2023, the Lombardy regional government launched a pilot program for primary cervical cancer screening, introducing self-sampling combined with HPV testing at one of the HPAs [31,32]. Following the COVID-19 pandemic, the General Directorate of Welfare of the Lombardy region initiated more robust and systematic monitoring of HPV screening, which was being implemented uniformly across the region for the first time. In 2023, a regional guidance document on cervical cancer screening was also published, serving as an additional tool to align the efforts of the various HPAs across the territory [33].

Regarding the new HCV campaign, the low level of advancement of HPAs on this issue strongly limited the evaluation opportunity. It is also necessary to define a specific procedure for each HPA and healthcare provider, with the definition of responsibilities.

The Lombardy region’s pioneering role in structuring the PRECEDE–PROCEED methodology for screenings in Italy stands as a significant strength. Additionally, the participation of the Control Agency and experts in both audit procedures and improvement plans, as well as in screening initiatives, further enhances its robustness. However, potential limitations include the lack of training among personnel from audited HPAs regarding the audit methodology. Furthermore, the COVID-19 pandemic resulted in the postponement of the audit cycle aimed at evaluating the implementation of improvement plans until 2022–2023, and the involvement of new personnel in the audit control cycle posed additional challenges.

The audit method, used as a quality assurance tool for screening, should also be mentioned. In fact, the use of this methodology made it possible to implement a multidimensional and multidisciplinary approach, encouraging collaboration between the different actors involved in all screening pathways and promoting dynamic interventions based on epidemiological, socio-psychological, administrative, political, and environmental factors, with guaranteed effectiveness and in line with national standards. On the other hand, unlike other internationally validated quality assurance tools [34,35], the implementation of an audit according to the PRECEDE–PROCEED model is not yet validated and remains an operator-dependent practice.

The future perspectives of the PRECEDE–PROCEED audit model entail its development, consolidation, and standardisation as a valuable tool for systematic application. This approach ensures not only a longitudinal assessment overview but also the creation of knowledge assets for personnel acquiring this methodology. Additionally, there is potential for the model to be exported and shared, facilitating its uptake and utilisation in various healthcare settings. Moreover, the evolution of critical thinking and self-assessment practices is envisioned, aligning with the goal of continuous improvement, and the model’s capacity to identify critical areas and enhance service provision underscores its potential to drive meaningful improvements in healthcare delivery.

## 5. Conclusions

From a process perspective, the Lombardy region’s experience positively confirms the potential of the PRECEDE–PROCEED model applied to a participatory audit process, both in relation to the improvement processes of regional stewardship and local capacity building.

The application of continuous audit cycles for the Lombardy region and the extension of the process to other regional and international realities could further verify the functionality of the model and the achievement of objectives in continuous qualitative and quantitative improvement.

## Figures and Tables

**Table 1:** Reference standards for epidemiological indicators, overall regional value, and values achieved by each HPA for the year 2019. The screening was offered to women aged 50–74 for mammography, men and women aged 50–74 for colorectal screening, and women aged 25–64 for cervical cancer screening. A list with explanations of the epidemiological indicators used is available in the Supplementary Materials.

Epidemiological Indicators	Standard (%)	Lombardy Region (%)	HPA 1 (%)	HPA 2 (%)	HPA 3 (%)	HPA 4 (%)	HPA 5 (%)	HPA 6 (%)	HPA 7 (%)	HPA 8 (%)
Coverage by invitation (colorectal) *	>95	100.7	112	107.2	99.5	87.1	106.1	96.9	92.6	88.2
Coverage by invitation (breast) *	>95	100	129.3	96.4	102.8	96	91.2	113.3	100.3	103.9
Coverage by invitation (cervical) *	>95	25.7	112.7	0.0	0.0	0.0	101.8	0.0	89.7	79.3
Coverage by examination (colorectal)	>50	43.6	51.2	59.1	42.2	42.7	35.9	62.8	46.5	36.8
Coverage by examination (breast)	>60	53.3	62.4	59.9	51.4	50.4	48.3	72	57	49.6
Coverage by examination (cervical)	>50	12.0	46	0.0	0.0	0.0	29.5	0.0	42.5	30
Pre-invitation exclusions (colorectal)	>2	5	3.4	10.3	4.5	0.2	0.6	4	2.5	16
Pre-invitation exclusions (breast)	>2	9	11.6	19.7	10.9	2.55	3.3	5.1	3.21	15
Pre-invitation exclusions (cervical)	>2	11.6	13.2	NA **	NA **	NA **	28.9	NA **	6.3	19.9
Recall rate (subsequent examinations, breast)	<5	5.3	6.4	4	4.8	6.9	4.8	7	4.9	4.2
Recall rate (first examinations, breast)	<7	10.6	12.8	8.3	9.3	13.7	11.1	13.6	9	7
Screen-detected breast cancers stage T2+ at subsequent rounds	<25	24	29.1	9.9	22	25	25.4	34.9	27	16.8
Complete colonoscopy rate	>90	93.1	88.2	95.8	95.5	95.3	92.3	89.9	90.9	98
Advanced lesions detection rate (colorectal)	>5	5.4	3.9	6.3	6.1	6.1	4.4	9	5	5.1
CIN2+ detection rate	>5	4.24	4.68	NA **	NA **	NA **	2.31	NA **	3.71	4.14
Inadequate tests (cervical)	<5	4.4	5.6	NA **	NA **	NA **	4.7	NA **	3.3	1.6
Waiting time between positive pap-test and colposcopy (within 56 days)	>90	55	38	NA **	NA **	NA **	96.4	NA **	23	70.5
Waiting time between the positive FIT and colonoscopy (within 30 days)	>90	44	36.9	64.5	65.7	52.7	25.3	48.4	39.5	21.2
Waiting time between positive mammography and further assessment (within 28 days)	>90	79.3	83	88.1	97.1	44.5	89.2	67.3	72.8	77.7
Waiting time between positive result and breast surgery (within 60 days)	>50	43.1	50	28.6	49.2	30.8	43.3	64.5	34.8	57

* Coverage by invitation could exceed 100% when the invited population was larger than the target group for that year. ** NA: not available, as the screening program was not activated in the HPA’s territory.

**Table 2:** Comparison of epidemiological indicators achieved at regional level and their differences in 2019 and 2022. Rows highlighted in green show improvements from 2019 to 2022, while those highlighted in red indicate a worsening of epidemiological indicators.

	Breast Cancer Screening		Colorectal Cancer Screening		Cervical Cancer Screening	
	2019	2022	2019	2022	2019	2022
Target population (N)	836,050	867,307	1,628,945	1,696,749	292,556	561,351
	+31,257		+67,804		+268,795	
Invitations (N)	772,842	827,138	1,567,021	1,732,035	225,317	308,942
	+54,296		+165,014		+83,625	
Coverage by invitation (%) (adjusted **)	100.0%	100.1%	100.7%	104.8%	25.7%	46.2%
	+0.1%		+4.0%		+20.5%	
Tests (N)	445,494	418,968	710,934	713,045	105,058	130,251
	−26,526		+2111		+25,193	
Adjusted participation rate (%) ***	67.5%	55.4%	46.5%	42.0%	49.7%	45.0%
	−12.1%		−4.5%		−4.7%	
Coverage by examination (%)	53.3%	48.3%	43.6%	42.0%	12.0%	20.1%
	−5.0%		−1.6%		+8.1%	
Assessments (N)	22,970	22,856	35,480	31,242	5008	2389
	−114		−4238		−2619	
Assessment/positivity rate (%)	5.3%	5.5%	5.0%	4.4%	4.8%	3.5%
	+0.2%		−0.6%		−1.3%	
Advanced pre-neoplastic lesions (N)	NA *	NA *	3806	2185	457	239
	-		−1 621		−218	
Advanced pre-neoplastic lesions detection rate (‰)	NA *	NA *	5.4‰	3.1‰	4.3‰	3.7‰
	-		−2.3‰		−0.6‰	
Cancers (N)	2 071	1 863	699	687	NA *	NA *
	−208		−12		-	
Cancer detection rate (‰)	4.7‰	4.5‰	1.0‰	1.0‰	NA *	NA *
	−0.2‰		0.0‰		-	

* NA: not available, as this was not an epidemiological indicator implemented for this screening program. ** Adjusted for the pre-invitation exclusions. *** Adjusted for the pre- and post-invitation exclusions.

## Data Availability

Data are available on request from the authors.

## Supplementary Materials

The following supporting information can be downloaded at: https://www.mdpi.com/article/10.3390/curroncol31100445/s1, Brief description and explanation for each of the screening indicators in Table 1 and Table 2.
