# Supplementary material for: Impact of PRECEDE–PROCEED Model Audits in Cancer Screening Programs in Lombardy Region: Supporting Equity and Quality Improvement
Source: Curr Oncol. 2024 Oct 3;31(10):5960–73. doi: 10.3390/curroncol31100445 (PMC11506358; doi:10.3390/curroncol31100445)
Supplement: Supplementary file 1 [file curroncol-31-00445-s001.zip › curroncol-3207906-supplementary.pdf]

**<u>Here's a brief description and explanation for each of the screening indicators of Table 1:</u>**

**Coverage by invitation**:
The proportion of the target population invited to participate in screening. It measures the outreach and engagement of eligible individuals in the program.

**Coverage by examination**:
The percentage of the target population that actually undergoes screening after being invited. This reflects the effectiveness of invitation efforts in prompting participation.

**Pre-invitation exclusions**:
The percentage of individuals excluded from the screening process before being invited, often due to prior medical conditions, recent screening participation, genetic predisposition programs, or other disqualifying factors.

**Recall rate**:
The proportion of screened individuals who are called back for further tests due to abnormal or unclear results. It reflects the screening's accuracy and sensitivity.

**Screen-detected breast cancers stage T2+ at subsequent rounds**:
The percentage of breast cancers detected in screening that are stage T2 or higher during follow-up rounds. This indicates the stage at which cancers are being caught, impacting prognosis.

**Complete colonoscopy rate**:
The percentage of individuals undergoing screening who complete the full colonoscopy. It assesses the effectiveness and thoroughness of colorectal screening.

**Advanced lesions detection rate**:
The proportion of screenings that result in the detection of advanced lesions, including cancers or high-risk adenomas, indicating the program's ability to identify serious conditions.

**CIN2+ detection rate**:
The percentage of cervical screenings that detect cervical intraepithelial neoplasia (CIN) grade 2 or higher, indicating the program's capacity to catch significant precancerous lesions.

**Inadequate tests (cervical)**:
The proportion of cervical screening tests that are considered inadequate or unusable, affecting the overall effectiveness of the screening process.

**Waiting time between positive Pap-test and colposcopy (within 56 days)**:
The average time between a positive Pap-test result and a follow-up colposcopy, ideally within 56 days. It reflects the efficiency of follow-up care.

**Waiting time between positive FIT and colonoscopy (within 30 days)**:
The time from a positive fecal immunochemical test (FIT) to a follow-up colonoscopy, with a target of 30 days, indicating timely follow-up for colorectal screening.

**Waiting time between positive mammography and further assessment (within 28 days)**:
The interval between a positive mammogram and further diagnostic assessments, aiming for a 28-day window to ensure prompt diagnosis.

**Waiting time between positive result and breast surgery (within 60 days)**:
The time from a confirmed positive result for breast cancer to surgery, with a target of 60 days, indicating prompt treatment initiation after diagnosis.

**Here’s a brief description and explanation for each of the screening indicators of Table 2:**

**Target population**:
The group of individuals eligible for screening that should be invited to participate.

**Invitations**:
The number of invitations sent to individuals in the target population.

**Coverage by invitation (%)**:
The proportion of the target population invited to participate in screening.

**Tests**:
The number of screening tests performed on individuals, indicating the total participation in the program.

**Adjusted participation rate**:
The percentage of individuals who underwent screening after adjusting for those who were excluded or deemed ineligible.

**Coverage by examination**:
The percentage of the target population that actually undergoes screening after being invited.

**Assessments**:
The number of follow-up second level diagnostic tests performed on individuals with positive or unclear initial screening results.

**Assessment/positivity rate (%)**:
The percentage of individuals who required further assessment after a positive or unclear screening result.

**Advanced pre-neoplastic lesions**:
High-risk lesions detected during screening that have the potential to develop into cancer, such as high-grade dysplasia or adenomas.

**Advanced pre-neoplastic lesions detection rate (‰)**:
The rate at which advanced pre-neoplastic lesions are detected per 1,000 screenings. It indicates the screening program’s ability to catch significant pre-cancerous changes.

**Cancers**:
The number of confirmed cancer cases detected during the screening process, indicating the program's effectiveness in identifying malignancies.

**Cancer detection rate (‰)**:
The number of cancers detected per 1,000 screenings. It provides an important measure of how effectively the screening program identifies actual cancers.

**Pre-invitation exclusions**:
Individuals excluded from the screening process before receiving an invitation, often due to prior medical conditions, recent screening participation, genetic predisposition programs, or other disqualifying factors.

**Post-invitation exclusions**:
Individuals excluded from screening after receiving an invitation, typically due to new medical information or conditions arising after the invitation was issued.
